# SmartCards as Analogous Tools to Operate Tablet Computers for Elderly—A Feasibility Study

**DOI:** 10.3390/healthcare9091198

**Published:** 2021-09-10

**Authors:** Stephanie M. Mueller, Bettina Göttke-Krogmann, Julia Kortus, Melanie Wiechmann, Michael Weber, Sarina Mozek, Wilfried Mau, Andre Golla, Gundula Huebner

**Affiliations:** 1 Institute of Psychology, Martin Luther University Halle-Wittenberg, Social Psychology, 06099 Halle (Saale), Germany; stephanie.mueller@psych.uni-halle.de (S.M.M.); kortus@burg-halle.de (J.K.); Wiechmann@psych.uni-halle.de (M.W.); 2 Burg Giebichenstein, University of Art and Design Halle, Textile Design, 06108 Halle (Saale), Germany; krogmann@burg-halle.de; 3 Johanniter-Unfall-Hilfe e.V., Landesverband Nord, 20097 Hamburg, Germany; michael.weber@johanniter.de (M.W.); Sarina.Mozek@johanniter.de (S.M.); 4 Medical Faculty, Institute of Rehabilitation Medicine, Martin Luther University Halle-Wittenberg, 06099 Halle (Saale), Germany; wilfried.mau@medizin.uni-halle.de (W.M.); andre.golla@medizin.uni-halle.de (A.G.); 5 Medical School Hamburg, Social Psychology, 20457 Hamburg, Germany

**Keywords:** digital communication, cognitive ability, social participation, elderly, UTAUT

## Abstract

Background: Older adults sometimes shy away from using modern digital communication devices due to lacking experience and fear of failure. Within the present project, SmartCards were developed as analogous means to operate tablet computers for older adults with little previous computer experience. We investigated whether the SmartCards-Tablet-System would (a) increase use of digital communication and (b) affect loneliness, autonomy, cognitive ability and wellbeing of the users. Methods: The suitability and acceptance of the system was evaluated during a three month trial period with seniors in retirement homes, seniors with home assistance care and a waiting control group. Results: Acceptance, duration of use and frequency of use were high in both experimental groups. Cognitive ability significantly improved after three months’ use in both experimental groups. Effects on loneliness, autonomy and wellbeing could not be observed. Discussion: Our results indicate that seniors are very much able and curious to use modern digital devices if the interface and hardware are adjusted to their needs and capabilities without being stigmatizing. The use of modern communication services and the World Wide Web can promote contact of seniors with their (younger) relatives.

## 1. Introduction

Today’s telecommunications are primarily conducted via digital channels (e-mail, WhatsApp, Facetime/Skype, voice message, etc.). Those older adults who are used to traditional forms of communication (telephone, postal service) and who lack experience in the use of computers and smartphones may shy away from trying out modern ways of communication [1,2]. The resulting gap between generations, the digital divide, adds to the already existing social isolation of older adults from immediate family members who more often than not live far away [3,4]. Internet-based devices (tablets, smartphones, etc.) bear the possibility to facilitate social contact between generations. Additionally, they could simplify access to useful information (news, weather, Wikipedia, etc.) and support services (activity monitor, food delivery, medicine reminder, etc.). Consequently, innovative technological solutions have the potential to promote and encourage autonomous living during older age. However, digital solutions are scarcely used by members of the target group. Despite introductory workshops and cross-generational skills trainings [5,6] the use of digital systems is perceived as difficult by many older adults. The main reasons are of both physical and psychological nature [1,2,5,6,7] they include overly difficult installation, set-up and operating procedures, lacking technical support during use and negative social influence. Other reasons for rejection are isolated applications with restricted range of functions, which limit their usefulness and compatibility [1,2,5,6,7].

The goal of the present project was to tackle these problems by involving representatives of the target group in the developmental process from the start. Target group involvement in the developmental process aims at increasing the perceived usefulness and consequently the acceptance of technology. The goal was to provide older adults with a simplified tablet device with only elementary functions and intuitive use, in a non-stigmatizing design. Devices for older adults (e.g., cell phones) tend to be chunky and often use pictures to indicate functions instead of words. These design choices render them easily distinguishable from conventional devices and tend to be perceived as infantilizing. To prevent this, we used an off-the-shelf tablet computer with enlarged icons and font sizes and reduced menu functions (no side swipe, no corner functions). To accommodate the mainly analog experiences of older adults, we additionally provided NFC cards (SmartCards) to operate the system. The present application (SmartCards) is specifically designed for those older adults who are interested in using new technology (tablets/smartphones) but lack experience and are concerned about not being able to operate the device. The analogous SmartCards are starter tools to become familiar with digital devices and to overcome insecurity. They may be used as temporary or additional self-help solutions to foster autonomy and self-efficacy until older adults feel comfortable and competent using a tablet PC. Each SmartCard represented one application (e.g., e-mail, skype contact to relatives). The cards were individually labeled and kept in a box like file cards. If a user wanted to use an application, he just had to plug-in the appropriate NFC card and the program started automatically. Target group representatives were involved in the design of the SmartCards, chose which functions and apps would be most desired, gave feedback on tablet interface and font choices and helped optimize the holding devices for tablet and SmartCards to fit the demands of advanced-age living and handling modalities. Additionally, experienced professionals from a local care service were included in the design process.

We propose that one relevant feature of successful target-group technology is age-appropriate user interface design. We suggest that SmartCards could possibly function as a low-tech solution to operate complex digital technology.

The current study will present the results of a 3-month trial period (henceforth: intervention), during which older adults at home and in retirement homes regularly used and evaluated the SmartCards-Tablet-System. Structured interviews were used to evaluate acceptance of the system and the perceived wellbeing, loneliness, cognitive ability and autonomy of the participants at the start of the intervention and after 3 months of use. We expect that engaging with the new technology will activate participants’ motivational and cognitive resources. Additionally, the use of modern communication strategies such as Skype and WhatsApp may bridge the gap to children and grandchildren. Social isolation and loneliness are associated with sleep disturbance, wellbeing and cognitive decline in ageing individuals [8,9,10]. Empowering older adults to use modern technology could pave the way towards more social interaction, emotional support and new cognitive demands. These in turn can contribute to the preservation of cognitive fitness and prolong self-determined and autonomous living in old age [11,12,13].

The objectives of the present study were (a) to assess factors that may explain intensity of usage of the SmartCards-Tablet-System and (b) to evaluate whether regular usage of the SmartCards-Tablet-System has an effect on measurements of loneliness, autonomy, cognitive ability and wellbeing. The theory of planned behavior (TPB) [14], the theory of reasoned action (TRA) [15] and the unified theory of acceptance and usage of technology (UTAUT) propose that the likelihood of technology use increases if participants (1) have a more positive attitude towards the use of the technology, (2) perceive themselves as capable of handling the technology and (3) are surrounded by family and friends who encourage use (subjective norm) [14,16,17]. We expected to find that participants who score high on these factors would use the SmartCards-Tablet-System more frequently and for longer durations (Hypothesis 1). In return, more intense use should be associated with more positive effects on the extent of loneliness, autonomy, cognitive ability and wellbeing. Structured interviews were conducted to assess these outcome variables.

Furthermore, we expected that participants still living at home (but with senior citizens’ home assistance service) would benefit the most from the use of the digital device (Hypothesis 2). Older persons who live in rural areas, especially those with mobility impairments but largely preserved cognitive abilities, are often isolated from social and cultural interactions due to limited public transportation and often great spatial distances to relatives. Passive past-times such as watching television are the predominant source of entertainment. In contrast, older adults in retirement facilities may have access to more frequent social interaction as well as cognitive stimulation. However, potentially greater cognitive as well as physical impairment may limit their ability to use and appreciate the features and programs of the SmartCards-Tablet-System to their full extent.

## 2. Methods

### 2.1. Participants

Thirty-three volunteers were recruited, but only thirty finished the study (25 female, 5 male; age range 58–95 years). Two participants dropped out for health reasons, and one participant passed away. All test values of these three participants were excluded from analyses.

Inclusion criteria were: living in a rural area, living in a retirement home or being treated by a senior citizens’ home assistance service and having little-to-no computer experience. Three groups of volunteers were recruited: two experimental groups (A. seniors receiving home assistance service; B. seniors living in retirement home) and one waiting control group (C. seniors living in retirement home). Participants were not randomized but were blind to outcome values. They were approached by their professional caregiver, informed about the studies’ parameters and asked if they wanted to take part.

As the different living arrangements of the groups suggest, participants differed significantly in their level of care (Chi^2^ = 8.58, *p* < 0.05), with the least care required for the participants in the home assistance group. Differences in age and number of children did not reach significance (age: Chi^2^ = 5.64, *p* = 0.060; children: Chi^2^ = 0.72, *p* = 0.698). For demographics, see Table 1.

All participants took part voluntarily and gave written informed consent. The study was conducted in accordance with the Declaration of Helsinki.

### 2.2. Materials and Procedure

**Figure 1:**
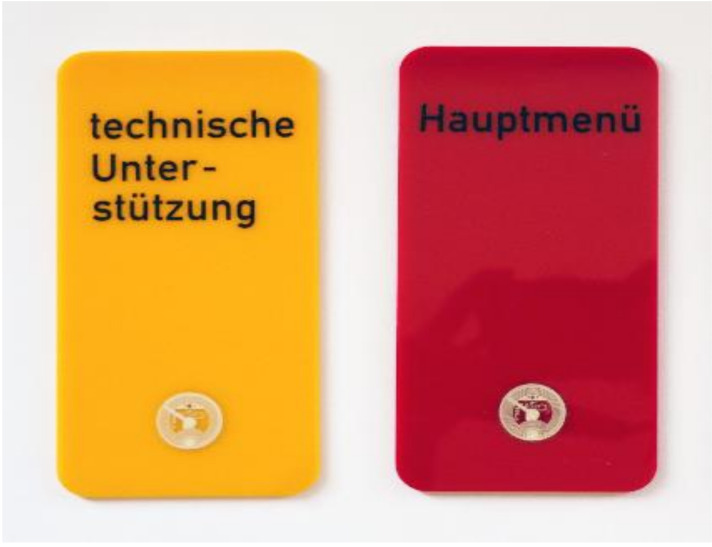
Two exemplary SmartCards with NFC chip on the bottom.

**Figure 2:**
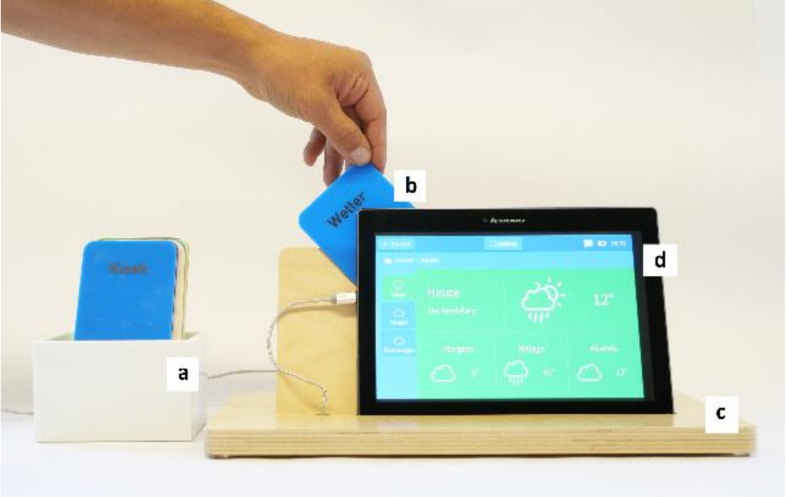
(**a**) SmartCards in card holder. (**b**) SmartCard in application rail. (**c**) Tablet holder made of wood. (**d**) Tablet PC with asina personal desktop. Photograph by Julia Kortus.

We used an off-the-shelf tablet computer with specialized user interface and apps (‘asina’ intuitive desktop for older adults by Exelonix GmbH, Dresden, Germany) that provided enlarged icons, font sizes and reduced menu functions (no side swipe, no corner functions). To accommodate the mainly analog experiences of older adults, we additionally provided NFC-based cards (SmartCards; Figure 1) to operate the system. Each card represented one application (e.g., e-mail). The cards were individually labeled and kept in a box like file cards (Figure 2). If a user wanted to use an application, he or she just had to plug-in the appropriate SmartCard, and the program started automatically. Alternatively, the touch screen could be used to operate the applications. App-usage frequencies via touch screen and SmartCards as well as duration of app use were tracked.

Participants were thoroughly instructed about the functions of the system. Each participant was given as much time as they needed to familiarize themselves with the system and ask all the questions they had. Asina personalized desktop had 12 icons that were linked to the following apps: e-mail, contacts/phonebook, photos, medicine reminder, notes, Internet Explorer, weather, calendar, kiosk, WhatsApp, Skype, and health diary. The kiosk app contained preferred links, e.g., local newspapers, meal delivery service, TV guide and games. Apps (e.g., local news, weather, meal plan) and contacts (e.g., Skype) were set up according to personal preferences and individual needs by a member of the study team. NFC cards were provided for all asina apps. Additional NFC cards were also provided for games (chess, sudoku), fitness videos (six different videos), TV guide and return to desktop.

The participants in the two experimental groups (A,B) used the system for three months. Technical support was provided by nursing staff if required. Structured interviews with rating-scale questions were used to assess pre- and post-test status of the outcome variables. At post-test, an additional questionnaire was used to evaluate the system. The control group was a waiting group. Participants in the control group were interviewed at the beginning of the study and then again after approximately three months. They did not receive any technical information or additional social interaction.

## 3. Study Design and Statistical Analyses

For this prospective feasibility study, a repeated-measures-between-subjects design was used. Feasibility studies are limited in their capacity to increase scientific knowledge. In the present case, the study explores suitable outcome measurements and the response/acceptance rates of the participants. Structured interviews were performed at baseline (pre-test) and after three months of using the device (post-test). Independent variables were experimental group, perceived behavioral control, attitude towards using the device and subjective norm. Dependent variables for Hypothesis 1 were frequency and duration of app use. Dependent variables for Hypothesis 2 were self-reported wellbeing, cognitive ability, loneliness and autonomy. For Hypothesis 2, mean values (pre and post) were calculated across clusters of items for each outcome variable. All pre–post comparisons for individual items are reported in Supplementary Materials. Due to limitations in normal distribution and group size, Wilcoxon signed rank and Mann–Whitney U tests were used for group comparisons. Alpha was set at 5%. SPSS software version 25.0 was used for data analysis.

## 4. Results

### 4.1. Descriptive Statistics

The participants in the home assistance group (A) significantly indicated the most confidence in their ability to use modern technical devices (Table 2). When asked about their intention to use the tablet and SmartCards, the participants in all three groups were equally eager to try the system. All participants felt that their friends and family would support and recommend the use of the technology. Participants in the home assistance group especially strongly agreed that their family would support the use of the device.

### 4.2. Hypothesis 1

We expected TRA and TPB indicators to show positive correlations with frequency and duration of app use. Contrary to our expectations, we did not find any associations between perceived behavior control, attitude towards use of the device, subjective norm and the actual duration and frequency of use (Table 3). Furthermore, duration and frequency of app use were statistically equal for participants in retirement homes (duration: median = 31 h 16 min, range = 95 h 29 min; frequency: median = 771, range = 2248) and under home assistance care (duration: median = 18 h, range = 921 h 55 min; frequency: median = 384, range = 1694; Mann–Whitney U test: duration: z = −0.898, *p* = 0.369; frequency: z = −1.777, *p* = 0.076).

Explorative qualitative analyses revealed that the most frequently used applications were telecommunication software (Skype and WhatsApp) and search tools (Google and Google Chrome). Nearly all participants also used the device to check the weather and read their favorite newspapers, as well as to use the TV program. Videos that were intended to inspire older adults to perform simple movement exercises and games that were intended to stimulate cognitive processes (chess, sudoku, memory) were hardly used by the participants.

### 4.3. Hypothesis 2

We expected to find favorable changes in the outcome variables for the experimental groups (Group A and B) but not for the control group when pre-test and post-test values were compared. Furthermore, we expected to find more pronounced improvements for the home assistance participants (Group A) than for participants in retirement homes (Group B).

The pre-test indicated just one significant difference between the groups, namely, wellbeing (Kruskal–Wallis H = 6.88, *p* < 0.05). Wellbeing was lowest in the home assistance group. No significant differences were found for self-reported cognitive ability (H = 4.91, *p* = 0.09), loneliness (H = 0.67, *p* = 0.72) or autonomy (H = 1.51, *p* = 0.47) at baseline.

**Figure 3:**
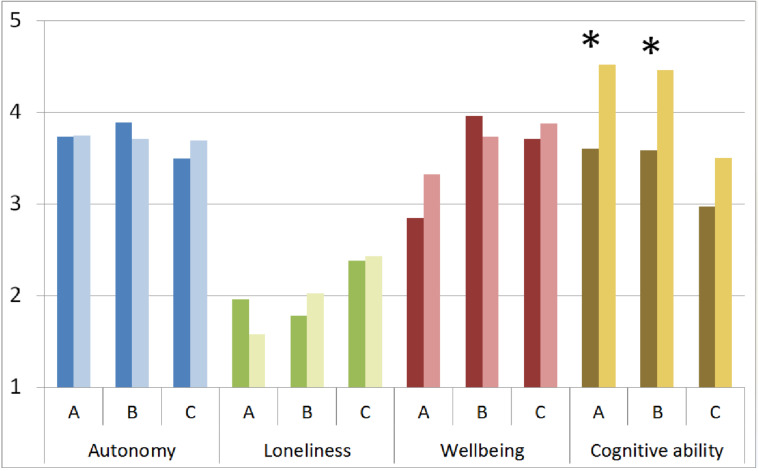
Mean differences between pre- and post-test cluster values of outcome variables. Dark colors: pre-test values, light colors: post-test values. A = home assistance service, B = retirement home, C = control group; Asterisks indicate significant differences.

Pre–post comparisons of the clustered outcome variables indicated significant differences in cognitive ability for both experimental groups (Table 4; Figure 3) but not for the control group. Clustered values of autonomy, loneliness and wellbeing did not differ between pre- and post-test in any of the groups. However, pre–post comparisons of all individual items (Supplementary Materials Tables S1–S4) also revealed significant differences in isolated items of the autonomy and loneliness clusters for the home assistance group (Group A). For groups B and C, none of the individual items reached significance. Even though these effects on the individual items are very small, they are in favor of our hypothesis that persons still living at home (home assistance) would benefit the most.

## 5. Discussion

Within the scope of the present study, we provided older adults living in rural areas with analogous tools (SmartCards) to operate a tablet PC. The goal was to reduce concerns about not being able to handle unfamiliar computer technology and to facilitate the use of modern digital communication and information services. We expected that SmartCards could help to overcome initial reservations concerning computer technology. The use of modern digital communication and information services could provide cognitive input and new challenges as well as foster social participation.

Contrary to our first hypothesis, we did not find an association between perceived behavior control, attitude towards use of the device, subjective norm and the actual duration and frequency of use. Furthermore, duration and frequency of app use were statistically equal for participants in retirement homes and under home assistance care. It is most likely that this result is due to a selection effect. Since all subjects took part voluntarily, their curiosity and willingness to try the device may have been above average. Another possibility is that, even though we did find differences between the groups in TRA and TPB indicators, there are three possible factors that could have mediated the effect: (1) participants received thorough information about and introductions of the device before they had to decide about participation; (2) the devices were delivered to them, personalized for them and had all their functions explained one-on-one; and (3) technical support (by caregivers and via hotline) was given throughout the study. These factors may have eliminated some of the concerns that often keep older adults from taking the leap to try modern communication technology: fear of not being able to use it and lack of technical support [5,6]. Therefore, even though we did find differences between the groups in perceived behavior control, attitude towards use of the device and subjective norm, the setting of the study may have counterbalanced these differences. Furthermore, the frequent use of the Google search engine by all participants seems to hint at the curiosity and willingness of the participants to explore and try out. More reluctant individuals might have kept to the pre-specified application links on the desktop and the SmartCards.

Additionally, this result could be interpreted as a first sign that the design and setup of the device were appropriate for the target group, which fostered acceptance and consequently the use of the device.

The second hypothesis was confirmed partially, insofar as significant differences between pre-test and post-test were found for cognitive ability in the experimental groups. This effect supports recent findings that showed that learning to use a tablet computer or a social networking site improves episodic and working memory as well as processing speed in older adults [18,19,20]. In line with our hypothesis, a more pronounced increase was found for the participants with home assistance than for the participants living in a retirement home. Self-reported measurements of loneliness, autonomy and wellbeing did not change after three months of using the device. However, the home assistance group did show a trend towards less loneliness post-intervention. One possible reason for the small effects may be that the questions may not have captured the fundamental changes that occurred. Anecdotally, many participants voiced their regret that the intervention period had to come to an end. Unstructured follow-up phone calls to the participating care services have revealed that now, several months after the study concluded, the majority of the participants in the experimental groups have acquired tablet computers of their own. This is in line with recent reports that possession of a smartphone increases internet literacy and internet use in elders [21] and that seniors prefer tablets over other mobile devices [22]. Other researchers report that learning to use a digital device such as a smartphone or tablet gave seniors a sense of accomplishment and satisfaction and gave participants a feeling of being able to face life more actively [23].

The results of the present study underline the importance of methodologically sound evaluation studies to examine the value and appropriateness of digital and AAL devices for the target group [24]. Our results show that a combination of introductory training sessions, the provision of SmartCards for easier access to favorite applications and a non-stigmatizing desktop design can build the foundation to overcome the widespread reluctance of older adults to use digital devices [25]. Low-level and inexpensive digital implementations like these bear the possibility to increase social participation as well as access to medical reminders and support. In the future, SmartCards may provide an analogous means to operate more complex systems such as AAL technology.

Limitations of the present study include small sample size, predominantly female gender, the field-study design with a selective sample, self-report data format and ambiguous duration of app use. Some participants did not close the apps after use. As a result, some of the app use duration times may not be due to actual use but due to the app running in the background. Future studies should prevent this weakness.

Since all outcome measurements were based on subjective self-evaluations, experimenter effects and Hawthorne effects cannot be ruled out. Future studies should include standardized tests to evaluate if the changes in cognitive functioning can be quantified.

## 6. Conclusions

Our results indicate that older adults are very much able and curious to use modern digital devices if the interface and hardware are adjusted to their needs and capabilities without being stigmatizing. In the future, SmartCards may provide an analogous means to operate more complex systems such as AAL-technology.

## Figures and Tables

**Table 1:** Demographics.

Groups		Age	Number of Children	Level of Care ^1^	Clock Drawing *
home assistance service A	*M*	81.75	1.75	1.29	2.17
	*n*	12	12	7	12
	*SD*	4.20	0.62	0.49	1.19
	Min	78	1	1	1
	Max	88	3	2	4
retirement home B	*M*	77.11	2.17	2.57	3.0
	*n*	9	6	7	9
	*SD*	11.53	0.98	1.13	1.32
	Min	58	1	1	1
	Max	95	4	4	5
control group C	*M*	86.11	1,89	2.29	3.56
	*n*	9	9	7	9
	*SD*	4.46	0.78	0.49	1.93
	Min	81	1	2	1
	Max	95	3	3	6

* Higher scores indicate more severe cognitive dysfunction. ^1^ Sign. difference in level of care, see text.

**Table 2:** Intention to use the device and expected competency with modern technology.

			Group					
			A	B	C	Chi^2^	Df	*p*
	I find it complicated to use new technology. I just can’t operate it.	*M*	2.92	3.11	4.44	8.09	2	<0.05
perceived behavioral control		*SD*	0.99	1.27	1.01			
	I would have liked to have learned to use a computer.	*M*	4.50	2.33	3.44	8.44	2	<0.05
		*SD*	1.17	1.80	1.94			
	I think I could handle technical devices well.	*M*	4.08	2.43	2.67	7.07	2	<0.05
		*SD*	0.99	1.27	1.66			
	It is a good idea to use the device.	*M*	4.83	4.67	3.80	3.70	2	0.16
		*SD*	0.39	0.71	1.64			
attitude towards the behavior	I am intending to try out the device extensively.	*M*	4.75	4.11	3.40	4.13	2	0.137
		*SD*	0.62	1.17	1.82			
	I like the notion to use the device.	*M*	4.67	4.11	3.00	4.06	2	0.13
		*SD*	0.65	1.05	2.00			
subjective norm	In the opinion of my family I should use technical assistance systems.	*M*	5.00	4.00	2.89	9.50	2	<0.01
		*SD*	0.00	1.73	1.83			
	In the opinion of my friends and acquaintances I should use technical assistance systems.	*M*	3.08	3.00	2.14	0.77	2	0.68
		*SD*	2.02	1.94	1.46			

Questionnaire used a 5 point Likert scale with the following possible answers: 1—“very much so”, 2—“mostly”, 3—“partly”, 4—“only a little”, 5—“not at all”; Group: A—home assistance service, B—retirement home, C—control group (retirement home).

**Table 3:** Correlation between TRA/TPB-scores, duration and frequency of app use.

			Frequency of Use *	Duration of Use *
	I find it complicated to use new technology. I just can’t operate it.	*r*	0.164	0.050
perceived behavioral control				
	I would have liked to have learned to use a computer.	*r*	−0.132	0.158
			
	I think I could handle technical devices well.	*r*	−0.217	−0.278
			
	It is a good idea to use the device.	*r*	0.117	0.140
			
attitude towards the behavior	I am intending to try out the device extensively.	*r*	0.145	0.173
		
	I like the notion to use the device.	*r*	0.364	0.204
			
	In the opinion of my family I should use technical assistance systems.	*r*	−0.128	0.081
			
subjective norm	In the opinion of my friends and acquaintances I should use technical assistance systems.	*r*	0.322	0.240
			

* all *p*-values > 0.10.

**Table 4:** Pre-post comparisons of cluster variables of perceived wellbeing, autonomy, loneliness, cognitive ability.

Group	Wilcoxon Signed Ranks Test	Autonomy	Loneliness	Wellbeing	Cognitive Ability
A	*z*	−0.714	−1.176	−1.426	−2.827
	*p*	0.475	0.240	0.154	<0.005
B	*z*	−0.740	−1.378	−0.816	−2.014
	*p*	0.459	0.168	0.414	<0.05
C	*z*	−0.170	−1.219	−0.138	−0.931
	*p*	0.865	0.223	0.890	0.352

## Data Availability

All relevant data is contained within the article and supplementary material.

## Supplementary Materials

The following are available online at https://www.mdpi.com/article/10.3390/healthcare9091198/s1, Table S1: Pre-post comparison of self-reported well-being. Table S2: Pre-post comparison of self-reported autonomy. Table S3: Pre-post comparison of self-reported loneliness. Table S4: Pre-post comparison of self-reported cognitive ability.
