# Supplementary material for: SmartCards as Analogous Tools to Operate Tablet Computers for Elderly—A Feasibility Study
Source: Healthcare (Basel). 2021 Sep 10;9(9):1198. doi: 10.3390/healthcare9091198 (PMC8466033; doi:10.3390/healthcare9091198)
Supplement: Supplementary file 1 [file healthcare-09-01198-s001.zip › healthcare-1281183-supplementary.pdf]

Supplementary materials

Table S1. Pre-post comparison of self-reported well-being.

		During the past two weeks I felt glad and in a good mood.	During the past two weeks I felt calm and at ease.	During the past two weeks I felt energetic and positive.	During the past two weeks I felt refreshed and well rested when I woke up.	During the past two weeks my day-to-day life was full of things that intrigued me.
Group A						
pre	*M*	3,25	2,17	3,08	2,33	3,55
	SD	,866	,937	,996	1,303	,934
post	*M*	3,58	2,83	3,50	2,92	3,75
	SD	1,240	1,193	1,243	1,881	1,765
	z	-1,26	-1,54	-1,29	-1,08	-,120
	p	,206	,123	,194	,277	,905
Group B						
pre	*M*	4,00	4,00	3,56	4,11	4,11
	SD	1,000	1,000	1,014	1,269	1,054
post	*M*	3,33	3,00	4,33	3,67	4,33
	SD	1,033	1,789	,816	1,506	1,211
	z	-1,34	-1,47	-1,00	-,447	-1,41
	p	,180	,141	,317	,655	,157
Group C						
pre	*M*	3,56	3,78	3,22	3,67	4,33
	SD	1,509	1,481	1,394	1,500	1,323
post	*M*	4,25	3,88	3,38	3,88	4,00
	SD	1,389	1,553	1,598	1,553	1,309
	z	-1,63	,00	,00	,00	-1,63
	p	,102	1,00	1,00	1,00	,102

Wilcoxon Signed Ranks Test
1 "very much so" 2 "mostly" 3 "partly" 4 "only a little" 5 "not at all"

Table S2. Pre-post comparison of self-reported autonomy.

		How much latitude do you have to make your own decisions?	To what extend can you influence your own future?	Do you think that the people around you respect your independence?	To what extend are you able to do what you wish to do?
Group A					
pre	*M*	4,42	3,17	4,25	3,08
	SD	,669	1,115	,754	1,165
post	*M*	3,42	3,33	4,25	4,00
	SD	1,165	1,497	1,215	1,206
	z	-2,326	-,398	-,159	-1,807
	p	,020	,690	,874	,071
Group B					
pre	*M*	4,11	3,22	4,00	4,22
	SD	,782	1,394	1,000	,833
post	*M*	4,00	3,00	4,50	3,33
	SD	1,095	1,265	,837	1,211
	z	-,816	,000	-1,000	-,850
	p	,414	1,000	,317	,395
Group C					
pre	*M*	4,11	2,33	3,88	3,56
	SD	,782	1,414	1,126	1,333
post	*M*	4,25	2,63	4,25	3,63
	SD	1,165	1,061	,886	1,061
	z	-,378	-,333	,000	-,378
	p	,705	,739	1,000	,705

Wilcoxon Signed Ranks Test
1 "very much so" 2 "mostly" 3 "partly" 4 "only a little" 5 "not at all"

Table S3. Pre-post comparison of self-reported loneliness

		I feel in unison with the people around me.	I have a lot in common with the people in my vicinity.	My interests and ideas are shared by the people around me.	If I want to find company I can and do.	There are people who I can exchange thoughts with.
Group A						
pre	*M*	1,73	2,55	3,00	1,36	1,18
	SD	,91	1,13	1,00	,67	,41
post	*M*	1,67	1,83	1,67	1,25	1,50
	SD	1,16	1,19	1,16	,62	,67
	z	-,367	-1,515	-2,026	-,378	-1,414
	p	,713	,130	,043	,705	,157
Group B						
pre	*M*	1,22	2,33	2,22	1,44	1,67
	SD	,67	1,23	1,30	1,01	1,12
post	*M*	1,33	4,00	3,00	1,83	2,00
	SD	,52	1,26	1,41	1,60	1,09
	z	-1,414	-,816	-,736	-1,342	-,816
	p	,157	,414	,461	,180	,414
Group C						
pre	*M*	2,22	2,44	2,67	2,33	2,22
	SD	1,48	1,81	1,80	1,41	1,48
post	*M*	2,50	3,00	2,50	1,88	2,25
	SD	1,51	1,41	1,69	1,36	1,58
	z	-1,134	-1,633	-,577	-1,000	-,707
	p	,257	,102	,564	,317	,480

Wilcoxon Signed Ranks Test
1 "very much so" 2 "mostly" 3 "partly" 4 "only a little" 5 "not at all"

Table S4. Pre-post comparison of self-reported cognitive ability

		Lately I often mix up names, phone numbers and dates.	Lately it has been harder to concentrate on a task.	Lately it is more difficult for me to follow other people's conversations.	Lately it has been more difficult to deal with problems.
Group A					
pre	*M*	3,33	3,25	4,25	3,58
	SD	1,30	1,60	1,06	1,44
post	*M*	3,92	4,42	4,92	4,83
	SD	1,68	1,24	,29	,39
	z	-1,150	-2,124	-1,807	-2,392
	p	,250	,034	,071	,017
Group B					
pre	*M*	3,33	3,22	4,44	3,33
	SD	1,41	1,20	,88	1,58
post	*M*	3,67	4,33	4,83	5,00
	SD	1,75	1,21	,41	,000
	z	-1,134	-,816	-1,633	-1,890
	p	,257	,414	,102	,059
Group C					
pre	*M*	2,89	2,78	3,78	2,25
	SD	1,83	1,92	1,64	1,49
post	*M*	3,13	2,88	4,13	3,88
	SD	1,46	1,55	1,36	1,46
	z	-,175	-,184	-,412	-1,841
	p	,861	,854	,680	,066

Wilcoxon Signed Ranks Test
1 "very much so" 2 "mostly" 3 "partly" 4 "only a little" 5 "not at all"
